# Fuel Cell Electrode Characterization Using Neutron Scattering

**DOI:** 10.3390/ma13061474

**Published:** 2020-03-24

**Authors:** Olaf Holderer, Marcelo Carmo, Meital Shviro, Werner Lehnert, Yohei Noda, Satoshi Koizumi, Marie-Sousai Appavou, Marina Appel, Henrich Frielinghaus

**Affiliations:** 1 Forschungszentrum Jülich, Jülich Centre for Neutron Science (JCNS) at MLZ, 85747 Garching, Germany; m.s.appavou@fz-juelich.de (M.-S.A.); marina.s.appel@gmail.com (M.A.); h.frielinghaus@fz-juelich.de (H.F.); 2 Forschungszentrum Jülich, Institute of Energy and Climate Research, IEK-14: Electrochemical Process Engineering, 52425 Jülich, Germany; m.carmo@fz-juelich.de (M.C.); m.shviro@fz-juelich.de (M.S.); w.lehnert@fz-juelich.de (W.L.); 3 Faculty of Mechanical Engineering, RWTH Aachen University, 52062 Aachen, Germany; 4 Ibaraki University, D302 IQBRC 162-1, Shirakata, Tokai-mura, Naka-gun, Ibaraki 305-8577, Japan; yohei.noda.77@vc.ibaraki.ac.jp (Y.N.); satoshi.koizumi.prof@vc.ibaraki.ac.jp (S.K.)

**Keywords:** SANS, WANS, HT-PEFC, electrode layer

## Abstract

Electrochemical energy conversion and storage is key for the use of regenerative energies at large scale. A thorough understanding of the individual components, such as the ion conducting membrane and the electrode layers, can be obtained with scattering techniques on atomic to molecular length scales. The largely heterogeneous electrode layers of High-Temperature Polymer Electrolyte Fuel Cells are studied in this work with small- and wide-angle neutron scattering at the same time with the iMATERIA diffractometer at the spallation neutron source at J-PARC, opening a view on structural properties on atomic to mesoscopic length scales. Recent results on the proton mobility from the same samples measured with backscattering spectroscopy are put into relation with the structural findings.

## 1. Introduction

Electrochemical energy conversion plays an important role for the current change in energy infrastructure. Fuel cells provide a clean way of electricity production from chemical energy, e.g., for the automotive sector or also stationary sector from kW to MW energy scales [1]. The environmentally friendly production of hydrogen gas would allow to store “green energy” and to convert it back in times of high demand or low electricity production. Electrolyzers and fuel cells are the components capable of converting between electricity and H${}_{2}$. The real world usage and operation of electrochemical energy converters depends strongly on the reliability of operation and the costs. A microscopic understanding of the transport processes is important in this context in order to optimize the different components of electrolyzers and fuel cells. Regarding fuel cells, many different technical realizations are available depending on the required application and available power, from solid oxide fuel cells for stationary applications, working at high temperatures, to polymer electrolyte membrane (PEM) fuel cells working below 100 ${}^{\circ }$C [2]. In this paper, high temperature polymer electrolyte fuel cells are discussed, which operate at temperatures of 120–180 ${}^{\circ }$C with advantages concerning water management and CO tolerance compared to PEM fuel cells working below 100 ${}^{\circ }$C [3]. At the heart of these devices is the ion conducting membrane, consisting of a polymer electrolyte membrane doped with phosphoric acid or potassium hydroxide, and the electrode layers around. A variety of techniques exist to characterize and analyze different aspects of the fuel cells, from the catalyst to the proton conducting membrane. Amongst the lab based techniques there is e.g., cyclic voltammetry [4] or specroscopic techniques such as IR adsorption spectroscopy for adsorption processes on Pt [5], or electron microscopy for the study of morphology changes in membrane electrode assemblies [6]. Previously, X-ray and neutron radiography have been applied to high temperature polymer electrolyte membrane (PEM) fuel cells and electrolyzers to gain insight into the hydrogen distribution across the area of the fuel cell in neutron radiography experiments [7] or across the membrane electrode assembly with high spacial resolution with synchrotron x-ray radiography [8]. A powerful suite of experimental techniques for characterizing electrodes and membranes on microscopic time- and length-scales are neutron scattering experiments [9]. The wavelength of neutrons allows to look at structures from atomic to macroscopic length scales with different scattering [10,11] and imaging techniques [12,13]. Moreover, the kinetic energy of neutrons allows to study proton diffusion processes on nanometer length scales [14]. The large penetration through materials allows even for “operando” experiments with neutron spectroscopy [15]. Such advanced characterization methods shall permit researchers to increase cell efficiencies, unveil degradation mechanisms, and fabricate the next generation of solid electrolytes for fuel cells and electrolyzers. Recently, the proton mobility inside the electrode layers of high temperature PEM electrodes has been studied with backscattering spectroscopy [16]. It could be shown that the mobility follows a jump diffusion model with traps.

In this manuscript, another important step for the characterization of electrode layers is given by presenting neutron scattering experiments where simultaneously data in the small angle and diffraction regime are taken on an electrode layer with different Pt loading with and without phosphoric acid. Scattering experiments probe the ensemble average of the sample and are in this sense complementary to the local real space information obtained with techniques such as electron microscopy. Finally, possible relations between the proton mobility and the structural properties of the electrode layer will be discussed.

## 2. Materials and Methods

### 2.1. Materials

In this contribution, electrode layers of HT-PEFCs are investigated. The gas diffusion elecrodes are prepared as described in Ref. [17,18,19] and for details we refer to this reference. In brief, Pt nanoparticles supported on carbon black with 20 and 60 wt% Pt were dissolved in water/propanol and mixed with PTFE (Dyneon TF5032Z, 24%) to an ink, which then was casted with a doctor blade technique onto a commercially available non-woven gas diffusion layer (GDL) with microporous layer (Freudenberg H2315C2), resulting in a catalyst loading of ≈1 mg/cm${}^{2}$. Phosphoric acid doping of the electrode layer was aiming to an amount of approximately 10 μL PA per cm${}^{2}$. A PA/ethanol solution (1:4 in volume) has been prepared and the corresponding amount of PA dropped onto the electrode layer.

### 2.2. Neutron Scattering

Neutron scattering experiments have been carried out at the iMATERIA beamline [20,21] at the Japanese spallation neutron source at J-PARC. The instrument covers a large range of scattering vectors, combining small angle and wide angle neutron scattering due to a large coverage with detectors and the inherent properties of a pulsed neutron source with a broad range of available neutron wavelengths with each pulse. The modulus q of the scattering vector ranges from q = 10${}^{-2}$ − 4 Å${}^{-1}$, covering length scales d = 2π/q from atomic distances to about 100 nm. Small angle neutron scattering (SANS) provides insight into mesoscopic length scales above 1 nm, averaged over the sample volume. Diffraction at larger angles can resolve atomic distances, in the case of such multicomponent systems such as the electrode layer, we do not use it here to characterize crystallographic structures by indexing a set of peaks, but to assign typical scattering vectors to peaks of a certain component. The different intensities can then be followed as a sign of changed crystallinity or changed structure.

### 2.3. Transmission Electron Microscopy

Dry membranes have been analyzed with a Transmission electron microscope (TEM). The images were obtained by using Thermo ScientificTM Titan 80-300 electron microscope equipped with a spherical aberration (Cs) corrector (CEOS) for the objective lens [22], and a JEM 2200 FS EFTEM instrument (JEOL, Tokyo, Japan) with zero-loss energy filtering. The sample preparation has been described in Ref. [19].

### 2.4. SANS Data Evaluation

A generic way of SANS data evaluation is provided by the Beaucage model [23,24]:

(1) $$I_{B}\left(q\right)=G\exp\left(-q^{2}R_{g}^{2}/3\right)+B{\left(erf{\left(qR_{g}/\sqrt{(}6)\right)}^{3}\right)}^{P}$$

with a characteristic radius of gyration $R_{g}$ and “Guinier-prefactor” *G*, and a power law decay with the Porod exponent *P* and scaling factor *B*, which can be written as

(2) $$B=\frac{GP}{R_{g}P}{\left(\frac{6P^{2}}{\left(2+P)(2+2P\right)}\right)}^{P/2}\Gamma \left(P/2\right)$$

with the Gamma function Γ(x). It yields a characteristic length scale via the radius of gyration, the power law decay *P* indicating the fractality of the structure.

The small angle part (corresponding to large structures) of the electrode layer has been evaluated with two levels of the Beaucage model plus a constant background which is mainly a result from isotropic incoherent scattering in the sample:

(3) $$I_{SANS}\left(q\right)=I_{B1}\left(q\right)+I_{B2}\left(q\right)+bgr$$

where bgr is a constant and $I_{B1/2}\left(q\right)$ are two instances of the Beaucage model, yielding two characteristic length scales $R_{1/2}$.

The diffraction part (WANS, wide angle neutron scattering) of the scattering curve with *q* > 1 Å${}^{-1}$ has been fitted with a sum of Gaussians:

(4) $$I_{WANS}\left(q\right)=\sum_{i}A_{i}\exp\left(-{\left(q-q_{i}\right)}^{2}/\left(2\sigma_{i}^{2}\right)\right)$$

The high-q regime has been fitted with *i* = 4 Gaussians, yielding the peak area, the position of the peak and the standard deviation for the different peaks. This rather phenomenological approach is sufficient in this case since the visible peaks are attributed to different materials in the sample.

The whole scattering curve can be described finally with the two contributions from SANS and WANS:

(5) $$I\left(q\right)=I_{SANS}\left(q\right)+I_{WANS}\left(q\right)$$

The available diffraction peaks are not sufficient for a crystallographic analysis and would mainly show the Pt structure as most crystalline component. We think therefore this procedure being adequate to identify the component and variations by analyzing the peak area of the respective contributions.

## 3. Results

**Figure 1:**
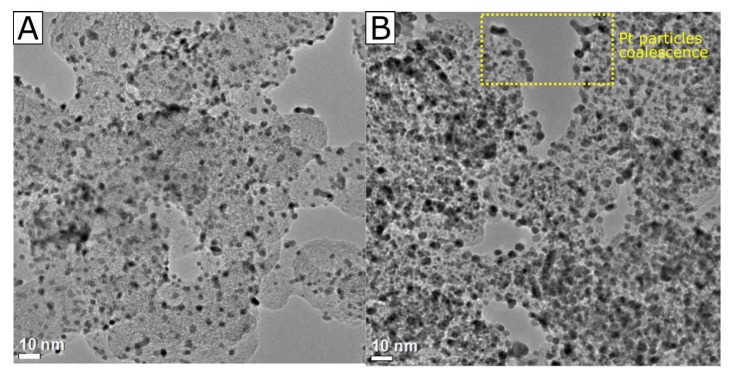
Bright-field TEM images of (**A**) 20%wt. Platinum supported on carbon black (Pt/C 20%), (**B**) 60%wt. Platinum supported on carbon black (Pt/C 60%), both catalysts commercially available from Johnson & Matthey. Pt/C 20% has an average particle size of 2.6 nm ± 0.5, and Pt/C 60% has an average particle size of 3.5 nm ± 0.5.

We first address the structure of the Pt/C catalysts using Transmission Electron Microscopy (TEM). Figure 1 shows the bright field TEM images of a 20 %wt. and a 60 %wt. platinum supported on carbon black from Johnson&Matthey. The mean particle diameter was 2.6 and 3.5 nm for the 20% Pt/C and 60% Pt/C respectively from TEM images. The polydispersity of the catalyst has been reported in Ref. [19] to be around 0.4 from small angle x-ray scattering (SAXS) measurements. The nanoparticles are homogeneously dispersed over the entire carbon surface, and only scattered agglomerates are observed. However, it is clearly noticed on the 60% Pt/C (Figure 1B) that small nanoparticles tend to coalesce into larger particles or form agglomerates. Nevertheless, we consider both catalysts options suitable for electrode fabrication and further characterization.

**Figure 2:**
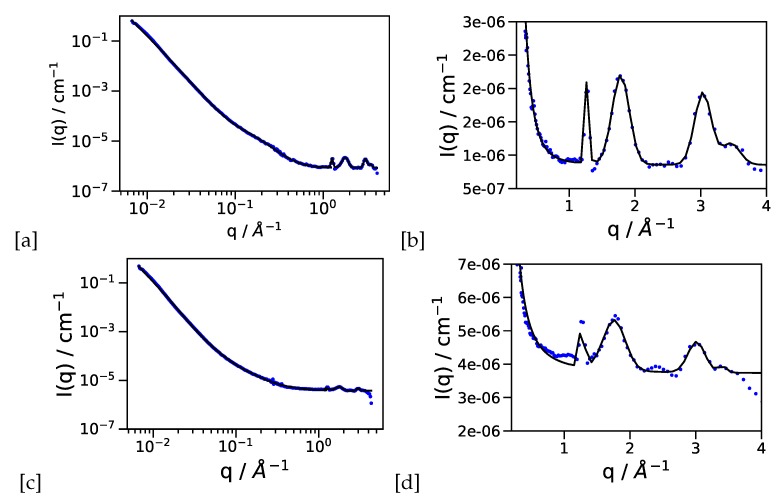
(**a**) SANS-WANS diffraction data for the empty 20% platinum containing electrode layer (**b**) only the high-q region shown on a linear scale (**c**) the same sample filled with phosphoric acid (**d**) high-q region of the phosphoric acid doped electrode. The hydrogen contents is responsable for the higher incoherent background.

While TEM gives an excellent impression on local length scales, scattering experiments provide the average information across the whole sample and are in this sense complementary. Secondly, also liquid components can be present in scattering experiments, therefore also phosphoric acid doped samples can be analyzed. Electrode layers with two different platinum catalyst loadings have been measured empty and loaded with phosphoric acid (PA). Figure 2 shows the results of the neutron scattering experiments with extended q-range for a catalyst loading of 20%.

## 4. Discussion

The structure of HT-PEFC catalyst layers has been analyzed with SAXS, SANS and transmission electron microscopy (TEM) with samples of the same kind (20% and 60% Pt) [19,25,26]. In these publications we have shown that the combination of different scattering methods highlights different parts of the sample. X-rays are mostly sensitive to heavy elements (as are electrons in TEM), thus highlighting the Pt catalyst, while neutrons have an irregular dependence of the scattering length on the element, with an additional isotope dependence, which allows for contrast variation by H-D exchange, e.g., with deuterated phosphoric acid. Especially in multicomponent systems this opens the way for highlighting different parts of the sample and getting in this way otherwise hidden details such as platinum particle size and agglomeration.

**Figure 3:**
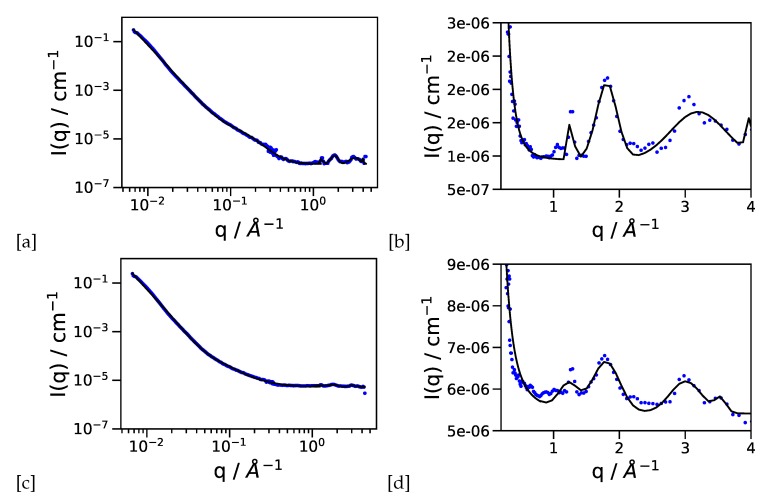
(**a**) SANS-WANS diffraction data for the empty 60% platinum containing electrode layer (**b**) only the high-q region shown on a linear scale (**c**) the same sample filled with phosphoric acid (**d**) high-q region of the phosphoric acid doped electrode. The hydrogen contents is responsable for the higher incoherent background.

Here we want to point out the experimental advantage of covering the q-range from 1–1000 Å${}^{-1}$ with the same sample. From the SANS regime (see Figure 2a,c and Figure 3a,c for 20% and 60% Pt loading respectively), we observe the characteristic length scales with the 2 instances of the Beaucage model presented in Table 1, with R${}_{g,0}$ of about 10 Å from the Pt particles, and a larger length scale R${}_{g,1}≃500$ Å of the supporting structure, with a fractal power law decay with an exponent *P* close to 4 indicating Porod scattering of a flat interface, i.e., no surface fractal structure. The phosphoric acid loaded samples show a slight increase in R${}_{g,0}$. Since the incoherent background is much higher due to the larger hydrogen contents, we think that the slope P has to be taken with care in this case.

At q > 1 Å${}^{-1}$ (see Figure 2b,d and Figure 3b,d for 20% and 60% Pt loading respectively with the results compiled in Table 2) we attribute the first peak (0) to the PTFE support present in the electrode (q${}_{0}≃$ 1.2 Å${}^{-1}$), the second peak (1) to the carbon support (q${}_{1}≃$ 1.8 Å${}^{-1}$), and peaks (2) and (3) to the Pt (111) and Pt (200) reflections. More Bragg peaks for a crystallographic analysis of the Pt particles are beyond the q-range of the experiment. We can see that the area of the peaks (A${}_{i}$) is approximately constant between empty and PA loaded membranes (with the largest uncertainty on peak (3) at the edge of the experimental range. The area can be compared therefore under different conditions of electrolyte (PA) loading. The ratio of carbon to platinum peak would be a measure of possible degradation of catalyst particles if electrodes after some time of operation are compared to fresh electrodes. The size change of the larger length scales of R${}_{g,0}$ is an indication of arrangement of phosphoric acid adsorbed onto Pt particles, especially for the 20% Pt loading, thus producing a larger spherical shell with a scattering length density (SLD) larger than the empty space around. With 60% Pt loading the size increase is smaller, since the phosphoric acid has to be distributed around significantly more Pt particles. The combination of R${}_{g,0}$ with the constant area of the Pt peak (2) suggests the adsorption of PA onto the Pt particles as a result of the broad range of length scales available in this experiment. Recent results from neutron backscattering spectroscopy on the diffusion of protons in the electrode layer showed that the protonic motion can be well described by a jump diffusion model with traps [16]. The parameters of the trapping model, i.e., trapping time and and mean squared distance between traps, depend on the structure of the catalytic layer and not e.g., on the doping level with phosphoric acid. The diffraction experiments presented here provide a consistent picture, that the phosphoric acid adsorption onto the Pt catalyst, which plays an important role concerning the proton diffusion in the catalytic layer, is also visible in the structure in this multicomponent system.

It has been observed also with X-ray absorption spectroscopy in Ref. [27] that phosphoric acid “poisons” the Pt catalyst surface, the specific ion adsorption depending on the applied cell potential and temperature, but generally the catalytic activity is reduced by anion coverage and blocking of active sites of the Pt particles.

Neutron scattering on length- and time-scales of nanometers and nanoseconds can complement electrochemical analysis of electrode properties such as electrocatalytically active surface area (ECSA) studies [28], where Pt alloys have been studied with respect to their catalytically active areas. An intensely discussed question is that of the long term stability of Pt containing catalysts with at the same time high catalytic activity [29,30]. The activity and stability of electrode layers can also be studied electrochemically with cyclic voltammetry [31,32]. Future investigations have to link the neutron scattering results with electrochemical investigations in order to achieve a thorough understanding of the structural influences and transport processes from atomic to macroscopic levels.

## 5. Conclusions

Neutron scattering provides a direct visualization window into the components of fuel cells and electrolyzers, here focused on electrode layers of high temperature PEM fuel cells. The heterogeneous structure over a wide range of length scales requires also experimentally that diffraction experiments are conducted over a broad range from atomic diffraction to mesoscopic SANS measurements (and possibly with radiography experiments to macroscopic length scales). This work showed that scattering experiments over a large range of length scales provide an insight into the individual components (catalyst particles, carbon structure) and investigate at the same time on larger scale the fractal structure of the electrode material and its evolution upon filling with electrolyte. The broad q-range helped in this case to complement and relate studies of local proton diffusion by neutron backscattering spectroscopy with structural indications of the phosphoric acid adsorption onto the Pt particles. Varying the contrast with H-D exchange allows to highlight different parts of the sample and distinguish structural properties of different materials, which will be a future continuation of the project. For example a contrast variation series with deuterated and protonated phosphoric acid could be made such that the surrounding PA has different contrasts to e.g., the platinum catalysts (SLD${}_{D_{3}PO_{4}}$ = 5.6 × 10${}^{-6}$ Å${}^{-2}$, SLD${}_{H_{3}PO_{4}}$ = 1 × 10${}^{-6}$ Å${}^{-2}$, SLD${}_{Pt}$ = 6.2 × 10${}^{-6}$ Å${}^{-2}$). In the SANS regime, such experiments have been presented in Ref. [25]. This is especially useful for such heterogeneous systems as electrode layers.

Covering atomic to mesoscopic length scales in one experiment as presented here has the unique advantage that structural data can be obtained on the same sample under the same conditions. This opens the path to heat cycles, where structural irreversible changes could occur, which is not possible if different instruments have to be used. Also for such heterogeneous systems one can guarantee in this case that for all length scales the sample conditions were truely identical. Also “operando” diffraction and SANS studies become feasable due to the use of a single neutron scattering instrument with this extraordinary covering of scattering vectors. As a future plan, experiments using dynamic nuclear polarization combined with SANS [33,34] are planned, where contrast variation is achieved in industrial type heterogeneous samples by polarizing the proton spin in the sample.

The microscopic view obtained here, and also local proton diffusion related to the structure as described in Ref. [16] can help understanding and finally optimizing macroscopic properties such as ion conductivity, catalyst loading or dispersion inside the electrode layer and may help in finding strategies for preventing or retarding degradation of the electrode layer.

In summary, neutron scattering experiments covering a large range in reciprocal space provide the potential to reveal structural properties on length scale from sub-nm to micrometers with unique contrast variation properties useful for samples with light elements and heterogeneous multi-component environments such a fuel cell electrodes. An exemplary experiment on a HT-PEFC electrode layer has been presented. Due to the different contrast and the ensemble averaging it is complemetary to electron microscopy.

## Figures and Tables

**Table 1:** SANS region with fit parameters for the evaluation with the Beaucage model with two characteristic length scales.

Parameter	Pure Electrode 20%	PA Loaded Electrode 20%	Pure Electrode 60%	PA Loaded Electrode 60%
G${}_{0}$ (cm${}^{-1}$)	7.334 × 10${}^{-6}$	4.060 × 10${}^{-5}$	9.197 × 10${}^{-6}$	1.684 × 10${}^{-5}$
G${}_{1}$ (cm${}^{-1}$)	2.442	1.392	1.902	1.121
bgr (cm${}^{-1}$)	2.331 × 10${}^{-7}$	9.733 × 10${}^{-7}$	2.664 × 10${}^{-7}$	1.620 × 10${}^{-6}$
R${}_{g,0}$ (Å)	9.945	2.908 × 10${}^{1}$	1.101 × 10${}^{1}$	1.663 × 10${}^{1}$
R${}_{g,1}$ (Å)	4.915 × 10${}^{2}$	4.537 × 10${}^{2}$	5.670 × 10${}^{2}$	5.027× 10${}^{2}$
P${}_{0}$	3.634	2.052	4.029	2.403
P${}_{1}$	3.847	4.054	3.79	3.972

**Table 2:** Diffraction region with q > 1 Å${}^{-1}$. Peaks were fitted with Gaussian curves. The parameters are peak area A${}_{i}$, peak postition q${}_{i}$, standard deviaiton of the Gaussian $\sigma_{i}$.

Parameter	Pure Electrode 20%	PA Loaded Electrode 20%	Pure Electrode 60%	PA Loaded Electrode 60%
A${}_{0}$ (cm${}^{-1}$)	1.259 × 10${}^{-6}$	1.431 × 10${}^{-6}$	7.926 × 10${}^{-7}$	6.270 × 10${}^{-7}$
q${}_{0}$ (Å${}^{-1}$)	1.271	1.272	1.270	1.226
$\sigma_{0}$ (Å${}^{-1}$)	2.969 × 10${}^{-2}$	3.758 × 10${}^{-2}$	3.386 × 10${}^{-2}$	1.439 × 10${}^{-1}$
A${}_{1}$ (cm${}^{-1}$)	1.344 × 10${}^{-6}$	1.505 × 10${}^{-6}$	1.161 × 10${}^{-6}$	1.213 × 10${}^{-6}$
q${}_{1}$ (Å${}^{-1}$)	1.784	1.760	1.807	1.797
$\sigma_{1}$ (Å${}^{-1}$)	1.308 × 10${}^{-1}$	1.675 × 10${}^{-1}$	1.557 × 10${}^{-1}$	2.075 × 10${}^{-1}$
A${}_{2}$ (cm${}^{-1}$)	1.086 × 10${}^{-6}$	9.214 × 10${}^{-7}$	7.273 × 10${}^{-7}$	7.673× 10${}^{-7}$
q${}_{2}$ (Å${}^{-1}$)	3.034	3.010	3.198	3.007
$\sigma_{2}$ (Å${}^{-1}$)	1.272 × 10${}^{-1}$	1.285 × 10${}^{-1}$	4.155 × 10${}^{-1}$	2.372 × 10${}^{-1}$
A${}_{3}$ (cm${}^{-1}$)	3.201 × 10${}^{-7}$	1.923 × 10${}^{-7}$	5.341 × 10${}^{-7}$	3.405 × 10${}^{-7}$
q${}_{3}$ (Å${}^{-1}$)	3.459	3.406	3.985	3.535
$\sigma_{3}$ (Å${}^{-1}$)	1.387 × 10${}^{-1}$	−6.162 × 10${}^{-2}$	5.440 × 10${}^{-2}$	8.120 × 10${}^{-2}$

## Abbreviations

The following abbreviations are used in this manuscript:

HT-PEFC	High Temperature Polymer Electrolyte Fuel Cell
PA	Phosphoric Acid
SLD	Scattering Length Density
TEM	Transmission Electron Microscopy
SANS	Small Angle Neutron Scattering
WANS	Wide Angle Neutron Scattering
